# Hypoxia-regulated carbonic anhydrase IX expression is associated with poor survival in patients with invasive breast cancer

**DOI:** 10.1038/sj.bjc.6603530

**Published:** 2007-01-09

**Authors:** S A Hussain, R Ganesan, G Reynolds, L Gross, A Stevens, J Pastorek, P G Murray, B Perunovic, M S Anwar, L Billingham, N D James, D Spooner, C J Poole, D W Rea, D H Palmer

**Affiliations:** 1 Cancer Research UK, Institute For Cancer Studies, University Hospital Birmingham, Edgbaston, Birmingham B15 2TT, UK; 2 Birmingham Women Hospital dgbaston, Birmingham, UK; 3 Liver Laboratories, University Hospital, Birmingham, UK; 4 Queen Elizabeth Hospital, Birmingham, UK; 5 Institute of Virology, Slovak academy of Sciences, Slovak Republic, Slovakia; 6 Walsgrave Hospital, Coventry, UK

**Keywords:** breast cancer, hypoxia, carbonic anhydrase (CA IX), prognostic marker

## Abstract

Tumour hypoxia is a microenvironmental factor related to poor response to radiation, chemotherapy, genetic instability, selection for resistance to apoptosis, and increased risk of invasion and metastasis. Hypoxia-regulated carbonic anhydrase IX (CA IX) has been studied in various tumour sites and its expression has been correlated with the clinical outcome. The purpose of this study was to investigate the correlation of CA IX expression with outcome in patients with invasive breast cancer. We conducted a retrospective study examining the effects of carbonic anhydrase IX (CA IX) on survival in patients with breast cancer. To facilitate the screening of multiple tissue blocks from each patient, tissue microarrays were prepared containing between two and five representative samples of tumour per patient. Immunohistochemistry was used to examine expression of CA IX in patients with breast cancer. The study includes a cohort of 144 unselected patients with early invasive breast cancer who underwent surgery, and had CA IX expression and follow-up data available for analysis. At the time of analysis, there were 28 deaths and median follow-up of 48 months with 96% of patients having at least 2 years of follow-up. CA IX was negative for 107 patients (17 deaths) and positive for 37 patients (11 deaths). Kaplan–Meier survival curves show that survival was superior in the CA IX-negative group with a 2-year survival of 97% for negatives and 83% for positives (log-rank test *P*=0.01). Allowing for potential prognostic variables in a Cox regression analysis, CA IX remained a significant independent predictor of survival (*P*=0.035). This study showed in both univariate and multivariate analysis that survival is significantly inferior in patients with tumour expressing CA IX. Prospective studies are underway to investigate this correlation in clinical trial setting.

Breast cancer represents a major public health problem, with more than 1 000 000 new cases and 370 000 deaths yearly worldwide. Despite pivotal developments in endocrine therapy and chemotherapy, resistance to therapy remains a key limitation in the management of invasive breast cancer. A fundamental difficulty in the understanding, prevention, and treatment of breast cancer is clinical heterogeneity between tumours. This is commonly manifested as variable responses to specific therapeutic regimens. Numerous studies indicate that this clinical heterogeneity reflects underlying molecular heterogeneity. Tumour hypoxia is a micro-environmental factor related to poor response to radiation and chemotherapy, genetic instability, selection for resistance to apoptosis, and increased risk of invasion and metastasis (Brizel *et al*, 1996; Graeber *et al*, 1996; Hockel *et al*, 1996; Reynolds *et al*, 1996; Kim *et al*, 1997). Tumour hypoxia has been studied in a variety of solid tumours and has been correlated with clinical outcome often using invasive monitoring of tissue oxygenation (Hockel *et al*, 1994; Brizel *et al*, 1995; Nordsmark *et al*, 1996; Fyles *et al*, 1998; Knocke *et al*, 1999; Fyles *et al*, 2002). Hypoxia has also been shown to have a prognostic impact in patients with breast cancer, head and neck cancer, and soft tissue sarcomas. The hypoxia-regulated protein carbonic anhydrase IX, CA IX, has been studied in various tumour sites and its expression has been correlated with clinical outcome (Chia *et al*, 2001; Loncaster *et al*, 2001; Kaanders *et al*, 2002; Swinson *et al*, 2003; Hussain *et al*, 2004). Carbonic anhydrase IX expression and its correlation with tumour oxygen measurements have previously been reported (Loncaster *et al*, 2001) and contradicted (Mayer *et al*, 2005). Hypoxia results in the upregulation of genes that facilitate anaerobic metabolism and promote tumour vascularisation (e.g. vascular endothelial growth factor, VEGF). Carbonic anhydrase IX is induced by hypoxia in a range of tumour cell lines in an hypoxia-inducible factor-1 (HIF-1)- dependent manner (Wykoff *et al*, Cancer Research, 2000), its role being to regulate tissue pH (Svastova *et al*, FEBS Lett, 2004). Studies have directly and indirectly validated the use of CA IX, as an intrinsic surrogate marker of hypoxia (Loncaster *et al*, 2001; Turner *et al*, 2002; Hoskin *et al*, 2003). In order to understand the contribution of hypoxia to tumour progression, resistance to treatment, and, in the future, to exploit differential expression of hypoxia-related factors in tumours *vs* normal tissue for therapeutic gain, we have examined the expression of CA IX in breast cancer and related this to clinico-pathological parameters and clinical outcome.

## MATERIALS AND METHODS

The study consists of 144 unselected patients with early invasive breast cancer who underwent surgery at the Queen Elizabeth Hospital Birmingham for which CA IX and follow-up data were available. To facilitate the screening of multiple tissue blocks from each patient, tissue arrays were prepared.

### Tissue microarray construction

Haematoxylin and eosin (H&E) sections from each case were screened by a pathologist and appropriate representative areas of tumour highlighted for sampling. Tissue cores for the construction of tissue microarrays (TMAs) were taken with a 3-mm skin punch biopsy needle (Stiefel Laboratories, UK) from the paraffin blocks that were used for histological diagnosis. The tissue cores were inserted in the holes of the rectangular carrier made of liver tissue, punched out by a 4-mm punch biopsy needle (Stiefel Laboratories, UK). The carrier facilitated the smooth cutting of sections with minimal artefacts in transition from paraffin to tissue.

Each carrier had a grid of 4 × 5 holes and, in addition to the breast tumour tissue samples, contained one core of normal breast tissue as a control. Four cores per sample, from multiple areas of the same tumour, were included in the TMA and embedded in different blocks at different positions on the grid for redundancy. After core insertion, tissue was re-embedded in paraffin. A small number of cores were damaged during TMA construction or subsequent methods and were labelled as noninformative. On an average, 83.4% of the cores, or 3.3 cores per specimen, were informative and were used in the analysis. (Murray *et al*, 2003; Tsoli *et al*, 2006).

### Immunohistochemistry

Sections 4 *μ*M were cut from each array block onto charged slides (Surgipath, UK) and heated for 1 h at 60°C. After deparaffinising and rehydration, sections were treated in 0.3% H_2_O_2_ in water to block endogenous peroxidase activity. Antigen retrieval was performed using the ALTER technique as previously described (Reynolds *et al*, 2002) in ethylene diamine tetraacetic acid (EDTA) buffer with Tween 20 overnight at 65°C. Following a brief wash in water, sections were placed onto a Sequenza (Shandon, UK) and washed in TBS (pH 7.6). A mouse monoclonal antihuman antibody (M75) raised to the external domain of CA IX (Pastorekova *et al* 1992) was applied at 1/100 dilution for 1 h (Table 1). After washing in TBS/Tween, primary antibody was visualised using Dako ChemMate Envsion detection kit (Dako, UK Ltd, Cat no. K5007) and Vector NovaRed chromagen (cat No SK-4800, Vector Laboratories, UK). Sections were then counterstained in haematoxylin, washed in water, rapidly dehydrated, placed into xylene and mounted in DPX. Negative controls consisted of sections processed in the same way but with omission of the primary antibody step.

### Evaluation of staining

Evaluation of immunostaining was performed on two separate occasions by one observer (SAH) and once by a pathologist (RG), both of whom were blinded to any other data. The whole of each section was subjectively assessed under light microscopy. For assessment of the level of CA IX expression, individual tumours were scored according to the level of staining in tumour cells. Staining of the biliary epithelial cells within surrounding liver (used to support the breast cores) served as the internal positive control (unpublished data). There was one score for the strength of staining (absent, weak, or strong, respectively) and one score for distribution pattern within the tumour stained (absent, focal/patchy, or diffuse). Sections where intra-observer or inter-observer error occurred for either of the scores were reviewed again by a pathologist (RG) and assigned a score that dictated which of the two original scores was recorded. In the event of all three pairs of scores differing, a consensus score was agreed upon after examination by both observers (SAH and RG) at a multiheaded microscope. Tumours were categorised into five patterns of expression of CA IX: tumour negative, weak diffuse (WD), weak focal (WF), strong diffuse (SD), or strong focal (SF). Expression of CA IX, whether strong or weak and focal or diffuse was grouped as positive.

### Statistical methods

Correlations between CA IX (positive or negative) and known prognostic variables of tumour size (numeric), tumour grade (low, intermediate, or high), axillary lymph node status (positive or negative), side of tumour (left or right), surgery type (mastectomy or WLE+ ALNC), vascular invasion (present or absent), ER status (positive or negative), DCIS grade (low, intermediate, high, or not seen), and the Nottingham Prognostic Index (numeric score derived from tumour size, histological grade, and axillary lymph node status; Galea *et al*, 1992) were explored by using *χ*^2^ and Fischer's exact tests for the categorical measures and *t*-tests for the numerical measures. Survival times were calculated as the date of primary tumour diagnosis to date of death, or date of censor, if alive. Survival curves were constructed using the method of Kaplan and Meier (1958), and the log-rank test (Peto *et al*, 1977) was used to assess any differences in survival time between levels of CA IX and levels of other known prognostic factors. Cox regression analysis was used to assess the association of CA IX with survival time alongside the other potential prognostic factors in a multivariate setting (Cox, 1972), with Wald tests used to report statistical significance of factors. Statistical analysis was carried out independently using SAS statistical software (SAS Institute, Cary, NC, USA).

## RESULTS

### Patient demographics

**Figure 1:**
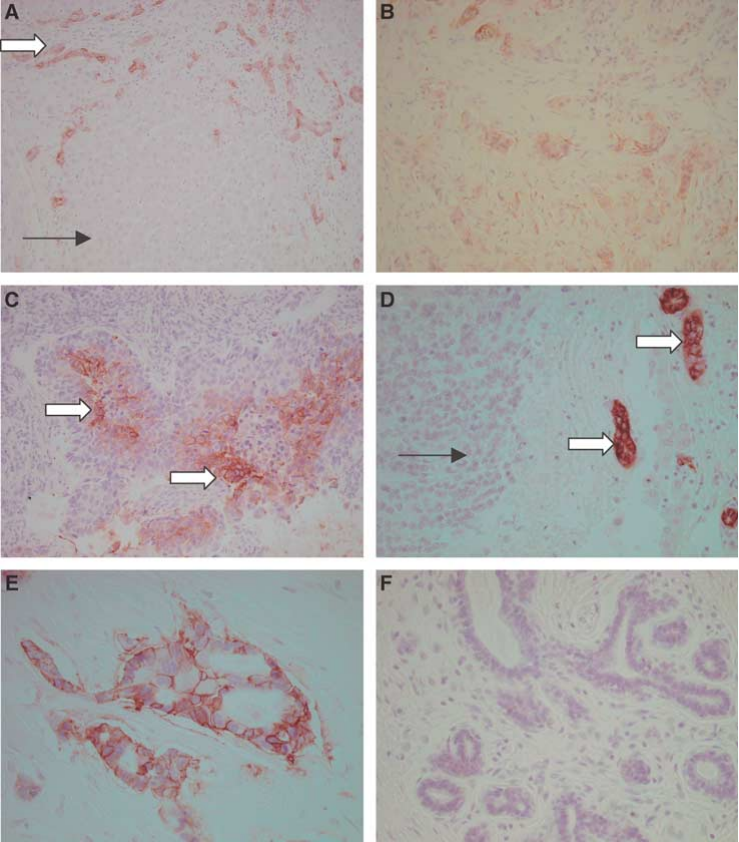
Carbonic anhydrase IX immunohistochemistry. (**A**) Weak focal staining. Some tumour cells are unstained (single arrow), while others show weak CA IX staining of cell membranes (block arrow) (magnification × 20). (**B**) Weak diffuse staining. All tumour cells show CA IX staining but the intensity is weak (× 20). (**C**) Strong focal staining. Groups of cells in the centre of tumour islands staining strongly on the cell membrane (block arrows) (× 20). (**D**) Strong focal staining. Some groups of tumour cells stain strongly (block arrows), while other areas of tumour show no staining (single arrow) (× 40). (**E**) CA IX staining is membranous (× 40). (**F**) Normal breast ducts and lobules do not stain with CA IX antibody (× 20).

The clinico-pathological demographics of the 144 women in this study are summarised in Table 2. Of these women, 131 (91%) had complete clinical data. Of particular note, the median age was 62 years, median tumour size was 2.2 cm, the proportion with positive axillary lymph nodes was 35%, and the proportion with ER-positive tumours was 72%. Carbonic anhydrase IX expression was detected in 37 of 144 cases (26%) (Figure 1) Expression of CAIX was always seen at the cell membranes. The distribution of this positive expression was largely in solitary or disparate foci of the examined tissue and was categorised as focal (Table 3).

### Correlation between CA IX expression and clinico-pathological features

There was no evidence that CA IX expression was associated with known prognostic variables, specifically tumour size (*P*=0.13), DCIS grade (*P*=0.89), axillary lymph node status (*P*=0.38), and ER status (*P*=0.11), side of tumour (0.50), surgery type (*P*=0.13), vascular invasion (*P*=0.59), tumour grade (0.40), and Nottingham Prognostic Index (*P*=0.39).

### Correlation between CA IX expression and survival

**Figure 2:**
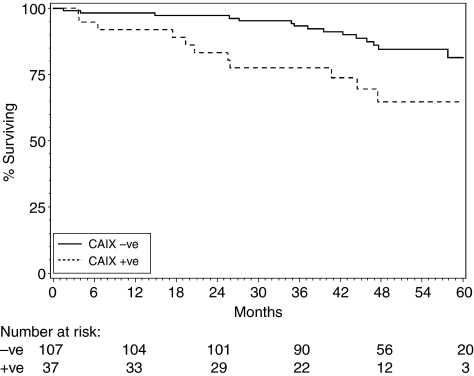
Kaplan–Meier survival curve by CA IX expression.

At the time of analysis, 28 of the 144 patients had died (17 and 11 in the CA IX negative and positive, respectively). Median follow-up for the alive patients was 48 months (range 5–71 months), with 96% having at least 2 years follow-up. In a univariate analysis, the data showed some evidence for vascular invasion, ER status, Nottingham Prognostic Index (NPI), and tumour grade to be prognostic for survival, although not all at a statistically significant level, possibly owing to the relatively small size of the study (Table 4). Survival was superior for the CA IX-negative tumours compared with CA IX positive (Figure 2), with a 2-year survival rate of 97 and 83% for CA IX-negative and -positive tumours, respectively. The hazard of death for patients with CA IX-positive tumours was approximately two and half times that for negatives (hazard ratio (HR)= 2.63; 95% confidence interval (CI) 1.21–5.70; log-rank *P*=0.01). In a Cox regression analysis, based on the 133 patients with complete data, even after adjusting for the NPI, CA IX remained significantly associated with survival (*P*=0.03). In fact, when adjusting individually for any of the prognostic variables in Table 1, CA IX always remained significantly associated with survival. Using the procedure of forwards stepwise selection and a significance level of 5% for inclusion of a variable in a model, it was found that the model best fit for accurately describing the variability in survival time should include the variables of vascular invasion (HR=2.33; 95% CI 1.01–5.56; *P*=0.049) and CA IX marker status (HR=2.43; 95% CI 1.07–5.53; *P*=0.035). The remaining variables of tumour size, tumour grade, nodal status, surgery type, ER status, and NPI did not reach the level of statistical significance required for inclusion in the model.

## DISCUSSION

Recent studies have demonstrated that many human tumours are hypoxic. This is likely to be due to compromised micro-circulation within a tumour. The clinical importance of tumour hypoxia is its association with a more aggressive malignant phenotype, increased risk of metastasis, and resistance to chemo- and radiotherapy (Brizel *et al*, 1996; Graeber *et al*, 1996; Hockel *et al*, 1996; Reynolds *et al*, 1996; Kim *et al*, 1997).

Carbonic anhydrase IX is induced by hypoxia in a range of tumour cell lines in an HIF-1-dependent manner (Wykoff *et al*, Cancer Research, 2000), its role being to regulate tissue pH (Svastova *et al*, FEBS Letters, 2004). Studies have directly and indirectly validated the use of CA IX as an intrinsic surrogate marker of hypoxia (Loncaster *et al* 2001; Turner *et al*, 2002; Hoskin *et al*, 2003).

In this study, we report that 37 of 144 unselected cases of invasive breast cancer express CA IX. Importantly, CA IX expression was not detected in normal breast tissue. Carbonic anhydrase IX expression was associated with a worse prognosis, even when controlling for other known prognostic factors.

These data are in general agreement with those published by Chia *et al* (2001). In that series of 103 women with breast cancer, CA IX was expressed in 48% of cases and was significantly associated with ER negativity, higher tumour grade and tumour necrosis. Multivariate analysis showed CA IX to be an independent predictor of overall survival with a HR of 2.61 (1.01–6.75). Similarly, another study (Vaupel *et al*, 1991) reported significant hypoxia in six out of 15 cases assessed by invasive computerised oxygen tension measurements.

In our series, the percentage of CA IX-positive tumours was lower (26%). This may be due, in part, to heterogeneity in CA IX staining both within and between individual tumours, which might lead to inaccuracy in estimating the number of positive and negative tumours. Additionally, this may be related to differences in technique and interpretation in nonstandardised immunohistochemistry assays. It was interesting to note that when adjusted individually for any of the prognostic variables, vascular invasion and CA IX expression always remained significantly associated with survival and the HR from our multivariate analysis was comparable to that found by Chia *et al.*

Hypoxia is reported to be an adverse prognostic factor in most human tumours. This study demonstrates that 26% of breast cancers are positive for CA IX expression. This information may have prognostic value in that CA IX expression is a predictor of poorer survival independently of other recognised prognostic factors. This information may, therefore, facilitate a more refined selection of patients for adjuvant treatment. By adding to established prognostic factors, CA IX expression may contribute to the identification of patients at greater risk of relapse who should be offered adjuvant treatment while sparing those whose prognosis is already good.

At the time of diagnosis for the patients in this series, Her-2 testing was not routinely performed. Thus, correlations between CA IX expression and Her-2 could not be made. Because of the prognostic importance of Her-2, this question must be addressed in future prospective studies in patients with known Her-2 status.

Furthermore, as hypoxia is related to resistance to chemotherapy and radiotherapy, CA IX expression may serve as a predictive factor to guide the selection of the most appropriate adjuvant treatment modality. Moreover, as hypoxia as a cause of treatment resistance can be effectively overcome by a variety of strategies, including high-oxygen-content gas breathing, blood transfusion, haemoglobin–oxygen affinity modifiers, and nicotinamide, the incorporation of these could be further investigated in patients with breast cancer.

Finally, the expression of CA IX in a number of breast tumours examined in this study, compared to the absence of CA IX in normal breast tissue, indicates that hypoxia and hypoxia-related gene expression may present a useful target for novel targeted therapies, for example drugs or gene therapy vectors that are specifically activated under hypoxic conditions. This study provides a rationale basis for the further study of these approaches in breast cancer. Randomised studies with translational end points are required to further elucidate the prognostic and predictive value of CA IX. Prospective study within the context of an adjuvant chemotherapy trial is underway to investigate and explore this correlation in clinical trial setting.

## Figures and Tables

**Table 1:** Antibody used

**Antibody**	**Code**	**Source**	**Dilution**
CA IX	M 75 monoclonal antibody to MN/CAIX	Institute of virology, Slovak, and Institute of molecular genetics Czech institute	1 : 100

CA IX=carbonic anhydrase IX.

**Table 2:** Patient demographics

**Characteristic**	**Statistic**
*Age*	
Median (IQR^a^)	62 years (52–74)
Range	(29–94)
	
*Surgery type*	
WLE+ALNC	65 (45%)
Mastectomy	79 (55%)
	
*Tumour size*	
Median (IQR)	2.2 cm (1.5–3.4)
Range	0.2–15
<2 cm	65 (45%)
>2 cm	79 (55%)
	
*Nodal status*	
Positive	51 (35%)
Negative	81 (56%)
Not known	12 (8%)
	
*Tumour grade*	
1	17 (12%)
2	78 (54%)
3	47 (33%)
Not known	2 (1%)
	
*DCIS grade*	
Low	5 (3%)
Intermediate	26 (18%)
High	63 (44%)
Not seen	50 (35%)
	
*Side of tumour*	
Left	70 (49%)
Right	72 (50%)
Not known	2 (1%)
	
*Vascular invasion*	
Present	38 (26%)
Absent	106 (74%)
	
*ER status*	
Positive	104 (72%)
Negative	40 (28%)
	
*NPI*	
<3.4	38 (26%)
⩾3.4	94 (65%)
Not known	12 (8%)

a IQR-interquartile range; NPI-Nottingham Prognostic Index.

**Table 3:** CA IX expression

**CA IX staining**	**Patient number**
Strong diffuse	0
Strong focal	13
Weak diffuse	1
Weak focal	23
Negative	107

CA IX=carbonic anhydrase IX.

**Table 4:** Univariate analysis of potential prognostic factors for survival

	**Hazard ratio**	**95% Confidence interval**	***P*-value from log-rank test**
Surgery type			
Mastectomy *vs* WLE	1.19	0.53–2.65	0.67
			
*Tumour size*			
⩾2 *vs* <2 cm	2.41	0.95–6.12	0.06
*Nodal status*			
			
Positive *vs* negative	1.42	0.63–3.22	0.40
			
Tumour grade			
3 *vs* 1, 2	1.80	0.80–4.02	0.15
			
*Side of tumour*			
Right *vs* left	1.16	0.52–2.57	0.71
			
*Vascular invasion*			
Present *vs* absent	2.56	1.11–5.89	0.03
			
*ER status*			
Positive *vs* negative	0.47	0.21–1.05	0.07
			
*NPI*			
⩾3.4 *vs* <3.4	2.39	0.82–6.99	0.11
			
*CA IX*			
Positive *vs* negative	2.63	1.21–5.70	0.01

CA IX=carbonic anhydrase IX; NPI=Nottingham Prognostic Index.
